# *Washingtonia filifera* seed extracts inhibit the islet amyloid polypeptide fibrils formations and α-amylase and α-glucosidase activity

**DOI:** 10.1080/14756366.2021.1874945

**Published:** 2021-01-25

**Authors:** Sonia Floris, Antonella Fais, Rosaria Medda, Francesca Pintus, Alessandra Piras, Amit Kumar, Piotr Marek Kuś, Gunilla Torstensdotter Westermark, Benedetta Era

**Affiliations:** a Department of Life and Environmental Sciences, University of Cagliari, Cagliari, Italy; b Department of Chemical and Geological Sciences, University of Cagliari, Cagliari, Italy; c Department of Electrical and Electronic Engineering, University of Cagliari, Cagliari, Italy; d Department of Pharmacognosy and Herbal Medicines, Wroclaw Medical University, Wrocław, Poland; e Department of Medical Cell Biology, Uppsala University, Uppsala, Sweden

**Keywords:** *Washingtonia filifera*, α-amylase, α-glucosidase, enzyme inhibition, islet amyloid polypeptide

## Abstract

*Washingtonia filifera* seeds have revealed to possess antioxidant properties, butyrylcholinesterase and xanthine oxidase inhibition activities. The literature has indicated a relationship between Alzheimer’s disease (AD) and type-2 diabetes (T2D). Keeping this in mind, we have now evaluated the inhibitory properties of *W. filifera* seed extracts on *α*-amylase, *α*-glucosidase enzyme activity and the Islet Amyloid Polypeptide (IAPP) fibrils formation.

Three extracts from seeds of *W. filifera* were evaluated for their enzyme inhibitory effect and IC_50_ values were calculated for all the extracts. The inhibition mode was investigated by Lineweaver-Burk plot analysis and the inhibition of IAPP aggregate formation was monitored.

*W. filifera* methanol seed extract appears as the most potent inhibitor of α-amylase, α-glucosidase, and for the IAPP fibril formation.

Current findings indicate new potential of this extract that could be used for the identification or development of novel potential agents for T2D and AD.

## Introduction

Consistent hyperglycaemia characterises type-2 diabetes (T2D), a condition caused by enzymatic degradation of carbohydrates and lipids in the cell, affecting glucose metabolism. Normalisation of blood glucose prevents hyperglycaemia and the development of diabetes complications. For diabetes patients, controlling postprandial hyperglycaemia is one of the main therapeutic targets. One way to do so is to reduce starch digestion rates by consuming low glycaemic index foods or intake of carbohydrate hydrolysing enzymes inhibitors^1–3^. The complex carbohydrates breakdown to glucose, through *α*-amylase and *α*-glucosidase enzymes, leads to the rapid absorption of glucose into the bloodstream leading to hyperglycaemia. Pancreatic *α*-amylase is a key enzyme in catalysing the initial step of complex carbohydrates (starch and glycogen) into shorter oligosaccharides, which are degraded to glucose by *α*-glucosidase in the small intestine^4^. These enzymes’ inhibition can reduce the high frequency of postprandial hyperglycaemia and prevent diseases resulting from blood glucose spikes in diabetes^5^.

Based on this strategy, acarbose, a potent inhibitor of *α*-amylase and *α*-glucosidase enzymes, has been approved as a prescription drug to treat T2D in some countries^6^. However, inhibition of these enzymes with commercial drugs such as acarbose results in gastrointestinal side effects that limit its use in a preventive approach^7^. For this reason, several research groups have investigated natural products with the inhibitory potential of key enzyme T2D-related, due to the highly abundant compounds in nature and for their promising biological activities^8–11^. Plants offer excellent alternatives to manage diabetes, as they are not only hypoglycaemic or insulin mimetic, but also prevent complications, while no synthetic drug provide these properties^12–14^.

The aggravation of T2D caused by chronic hyperglycaemia and subsequent increase of reactive oxygen species (ROS) leads to an augmentation of insulin resistance. The inhibitors with antioxidant activity might have significant therapeutic potentials because the oxidative injury appears to be a fundamental mechanism underlying a few human disorders, including diabetes^15–17^.

IAPP is released together with insulin in response to metabolic stimuli, supporting a role for IAPP in glucose metabolism, possibly as an insulin counter-regulatory hormone^18^. Post-mortem, IAPP amyloid is present in 96% of individuals with T2D^19^, and recently detected in pancreas biopsies from patients newly diagnosed with Type 1 diabetes (T1D)^20^. Oligomers that occur early in the amyloidogenesis are cytotoxic to β-cells^21^^,^^22^, and the amyloid deposits disrupt islet architecture and interfere with cell-cell signalling. For this reason, more anti-diabetic drugs with complementary mechanisms of action should be developed.

Furthermore, Madhusudhanan and co-workers^23^ have highlighted the correlation between T2D and Alzheimer’s disease (AD). AD and T2D are two of the most prevalent diseases in the elderly population worldwide. The pathological hallmark common to both diseases is local amyloid deposition, where aberrant aggregation of amyloid-beta (Aβ) and IAPP is implicated in AD and T2D, respectively. It is also evident that patients with T2D are at a higher risk of developing AD, which is why the concept of metabolism-dependent neurodegeneration mechanisms is gaining importance. Moreover, the phenomenon of oxidative stress is known to play an important role in both AD and T2D due to the neurodegeneration and diabetic complications, respectively, promoted by ROS.

*Washingtonia filifera* (Linden ex André) H.Wendl. ex de Bary belongs to the palm family that includes plant species with wide application in human food, some of which may also be of pharmacological interest^24^. The nutritional composition of fruits, of *W. filifera* have been analysed resulting a relevant source of dietary oils and a higher concentration of carbohydrates^25^^,^^26^. The aerial part of *W. filifera* were also investigated for their phytochemicals and antioxidant activities showing the presence of different flavonoids.

A previous report from our laboratory revealed the chemical composition, anticholinesterase, and antioxidant activities of *W. filifera* seed extracts, which resulted in being a promising source of bioactive compounds with inhibitory potential^27^. Encouraged by our recent finding, in this work the inhibitory properties of the *W. filifera* seed extracts on *α*-amylase, *α*-glucosidase and IAPP fibrils formation, in relation to T2D, have been evaluated.

## Materials and methods

### Plant material

The fruits of *W. filifera* were collected in Tunisia in the areas of Gabès (G) and Sousse (S). The fruits of *W. filifera*, were separated as pulp and seeds. The last were crushed separately then macerated in different solvent; precisely, 25 g of seeds were extracted in 100 mL of water (AE, aqueous extract), ethanol (EE, ethanol extract) or methanol (ME, methanol extract) for 72 h, at room temperature under continuous stirring. After filtration and centrifugation at 10,000 rpm, ethanol and methanol extracts were concentrated under vacuum using a rotary evaporator while the obtained aqueous extracts were then lyophilised for further analysis. In order to perform the characterisation of phenolic composition, fats and other non-polar compounds where removed by treating the extracts with *n*-exane as following: 10 mL of *n*-hexane were added to 200 mg of extract, filtered under vacuum with Bruckner, and the control of filtrate on TLC.

### UHPLC-ESI-QqTOF-MS analyses

Kinetex 150 × 2.1 mm ID 2.6 µm, options: 1) F5 (pentafluorophenyl) 2) C18 was used (injection volume 5 µL, 0.5 mg/mL). The elution gradient established was isocratic 0–10% B over 0.8 min, 10–14% B over 14 min, 14–15% B over 23.5 min, 15–60% B over 28.7 min, 60–100% over 37 min and re-equilibration of the column, where the elution buffers were formic acid 0.1% on aqueous solution (A) and formic acid 0.1% on acetonitrile solution (B). Double online detection was carried out as in the DAD using 210, 280, 330 and 370 nm as the preferred wavelengths. Compact QqTOF-MS (Bruker Daltonics) was operated in negative and positive mode and calibrated with the sodium formate clusters. The calibration segment was introduced at the beginning of every run. The main MS parameters were following: scan range 50–1400 m/z, nebuliser pressure 1.5 bar, dry gas (N2) 7.0 L/min, temperature 200 °C, capillary voltage 2.2 kV (negative mode) 4.5 kV (positive mode), ion energy 53 eV, collision energy 8 eV. The analysis of the obtained mass spectra was carried out using Data Analysis software (Bruker Daltonics).

### α-Amylase activity

A reaction mix containing 60 µL of 50 mM sodium phosphate buffer at pH 7.0, 20 µL of 1 M NaCl and 40 µL of *α*-amylase from porcine pancreas (1 mg/mL) (EC 3.2.1.1) was used. The solution was incubated in the absence or presence of seed extracts at 37 °C for 10 min. After incubation, 80 µL of a 2.5 mM 2-chloro-4-nitrophenyl-α-D-maltotrioside (CNPG3) solution was added as the substrate and 2-chloro-nitrophenol released by the enzymatic hydrolysis was monitored at 405 nm. Different concentration ranges were used for the assays: 0–12 µg/mL for alcoholic extracts and 0–40 µg/mL for aqueous extracts.

### α-Glucosidase activity

*α*-Glucosidase assay was performed as described by Fais et al.^28^. The enzyme (0.125 U/mL) (EC 3.2.1.20) solution was dissolved in 0.1 M sodium phosphate buffer (pH 6.8). Twenty microliters of test samples at various concentrations (0–2 µg/mL) were mixed with the enzyme solution in microplate wells and incubated for 15 min at 37 °C. Subsequently, 20 µL of 5 mM *p*-nitrophenyl *α*-D-glucopyranoside (pNPG) solution in 0.1 M phosphate buffer was added. After incubation at 37 °C for 15 min, the reaction was terminated by the addition of 50 µL of 0.2 M sodium carbonate solution. *α*-Glucosidase activity was determined spectrophotometrically at 405 nm on 96-well microplate reader by measuring the amount of *p*-nitrophenol released. DMSO control was used whenever required and the final concentration of DMSO was maintained below 8% v/v, which was found that it is not affecting the enzyme activity. Acarbose was used as a reference inhibitor for both enzymes. The IC_50_ values, the concentration giving 50% inhibition of enzyme activities, were determined by the interpolation of dose–response curves.

Kinetic of *α*-amylase and *α*-glucosidase inhibition was determined by the Lineweaver-Burk double reciprocal plot. The assays were performed increasing the concentration of the respective substrates in the absence and presence of the extracts at different concentrations.

The equilibrium constants for binding with the free enzyme (K_I_) and with the enzyme-substrate complex (K_IS_) were obtained either from the slope or the vertical intercepts plotted versus inhibitor concentration, respectively.

### ThT binding assay

IAPP stock solutions were prepared by dissolving 2 mg of synthetic amylin in 250 µL (2 mM) of hexafluorisopropanol (HFIP). This stock solution was stored at −20 °C. All solutions for these studies were prepared by adding a PBS buffered (1 mM) thioflavin-T solution to IAPP peptide (in lyophilised dry form) immediately before the measurement. The final concentration of IAPP solution was 40 µg/mL. When compounds were present, the IAPP to compound ratio was at 1:10, 1:5, 1:1, 1:0.1 and 1:0.01 by weight (400 µg/mL, 200 µg/mL, 40 µg/mL, 4 µg/mL, 0.4 µg/mL). 0.5% DMSO was present in the solution. ThT fluorescence was monitored at 480 nm with 440 nm excitation at 37 °C on a FLUOstar Omega microplate reader. The experiments performed as sextuplicates were repeated three times.

### Congo red stain

Droplets (5 µL) of the peptide solutions were placed three times on the same spot (15 µL) on a glass slide. When dried, the slides were put in Congo B solution for 5 min, twice in absolute ethanol for 10 s, twice in xylene for 1 min and then mounted.

### Negative stain

A drop of 5 µL of the samples from the plate suspended in 15 µL of distilled water was applied on to the grids strengthened with a carbon coating. The excess was then drawn off with filter paper and the grid was air dried. A drop of 50% uranyl acetate and 50% absolute ethanol was applied to the grids for 30 s. The excess was again drawn off with filter paper and the grid finally was air dried.

#### Molecular docking

The three-dimensional (3 D) structure of IAPP^29^ (PDB id: 2L86) and α-amylase^30^ (PDB id: 1DHK) were obtained from protein data bank. However, due to the unavailability of the experimental 3 D structure of an α-glucosidase protein from Saccharomyces cerevisiae, we performed a template-based homology modelling using Swiss-model web server^31^ with 3 D reference structure of isomaltase from Saccharomyces cerevisiae^32^ (PDB id: 3AJ7) having 72% sequence identity^33^ with the target protein structure. The predicted protein tertiary structure model was evaluated by local quality estimates^34^ and Ramachandran plot of the dihedrals. The 3 D structures of the ligands were obtained using open-babel software^35^; the details of ligand preparation have been described in our previous studies^36^^,^^37^. The docking experiment to generate and predict best protein-ligand complex pose was performed using a COACH-D server^38^.

### Data analysis

All experiments were performed in triplicates and the data were expressed as mean ± standard deviation (SD). Statistical differences were evaluated using GraphPad Prism software version 8 (San Diego, CA, USA). The comparison between groups was conducted by one–way analysis of variance (ANOVA) followed by the Tukey Multiple Comparisons Test. A *p* values of less than 0.05 was considered statistically significant.

## Results and discussion

### UHPLC-DAD-ESI/MS analysis

In our previous work we have analysed the alcoholic extracts of *W. filifera* seeds, using HPLC–DAD–ESI/MS. We have highlighted that the composition of these *W. filifera* seeds consisting in flavan-3-ol. B-type procyanidin dimers (B1-B4) were among the main phenolic compounds in the extracts of *W. filifera* seeds^27^. In this study, through UHPLC-DAD-ESI/MS analysis, three phenolic compounds have been identified, catechin, protocatechuic acid, and *p*-hydroxybenzoic acid, in addition to those identified in our previous report.

### α-Amylase and α-Glucosidase inhibition

The ability of the alcoholic and aqueous extracts of *W. filifera* seeds to restrict *α*-amylase and *α*-glucosidase activities was evaluated and the results are reported in Table 1.

**Table 1.** IC_50_ values of the aqueous (AE), ethanolic (EE) and methanolic (ME) extracts from *W. filifera* collected in the areas of Sousse (S) and Gabès (G) against *α***-**glucosidase and *α*-amylase.

Extracts	IC_50_ µg/mL	IC_50_ µg/mL
	*α*-glucosidase	*α*-amylase
EEG	1.54 ± 0.11^a^	11.33 ± 1.99^a^
EES	0.72 ± 0.41^a^	3.73 ± 0.45^b^
MEG	0.53 ± 0.014^a^	6.75 ± 0.11^b,c^
MES	0.88 ± 0.028^a^	2.39 ± 0.23^b^
AEG	1.63 ± 0.23^a^	48.32 ± 1.32^d^
AES	0.82 ± 0.085^a^	25.82 ± 0.13^d^
Acarbose	90 ± 7.3^b^	8.04 ± 0.65^a,c^

Mean values in the same column having different letters are significantly different (*p* < 0.05).

All extracts exhibited potent inhibitory activity on *α*-glucosidase. The IC_50_ values are statistically lower than that of the reference inhibitor, ranging from 0.53 ± 0.014 to 1.63 ± 0.23 µg/mL. In particular, the *α*-glucosidase inhibitory activity of the MEG was found to be ∼170 times more active than acarbose.

As can be observed from Table 1, some extracts inhibited *α*-amylase with a higher potency than the standard acarbose. MES showed the highest *α*-amylase inhibitory activity with IC_50_ 2.39 ± 0.23 µg/mL. In our previous study^27^ we have identified in the B-type procyanidin dimers (B1-B4), the main phenolic compounds in the alcoholic extracts of *W. filifera* seeds. In addition to those, in this study, three phenolic compounds have been identified, catechin, protocatechuic acid, and *p*-hydroxybenzoic acid.

In literature exist^1^^,^^39^^,^^40^ many studies that have reported inhibition mode or IC_50_ for metabolites such as procyanidin B-type dimers, catechin, protocatechuic acid, *p*-hydroxybenzoic acid, against α-glucosidase and α-amylase enzymes. It is worth pointing out that each compound showed an IC_50_ value higher than that shown by the MES.

Compared to the MES extract, the type B procyanidin dimer has higher IC_50_ values towards α-glucosidase and comparable for α-amylase^41^.

The synergic action of these compounds could contribute to explain the significant inhibition of the *W. filifera* methanolic extract against *α*-amylase and *α*-glucosidase.

**Figure 1.**
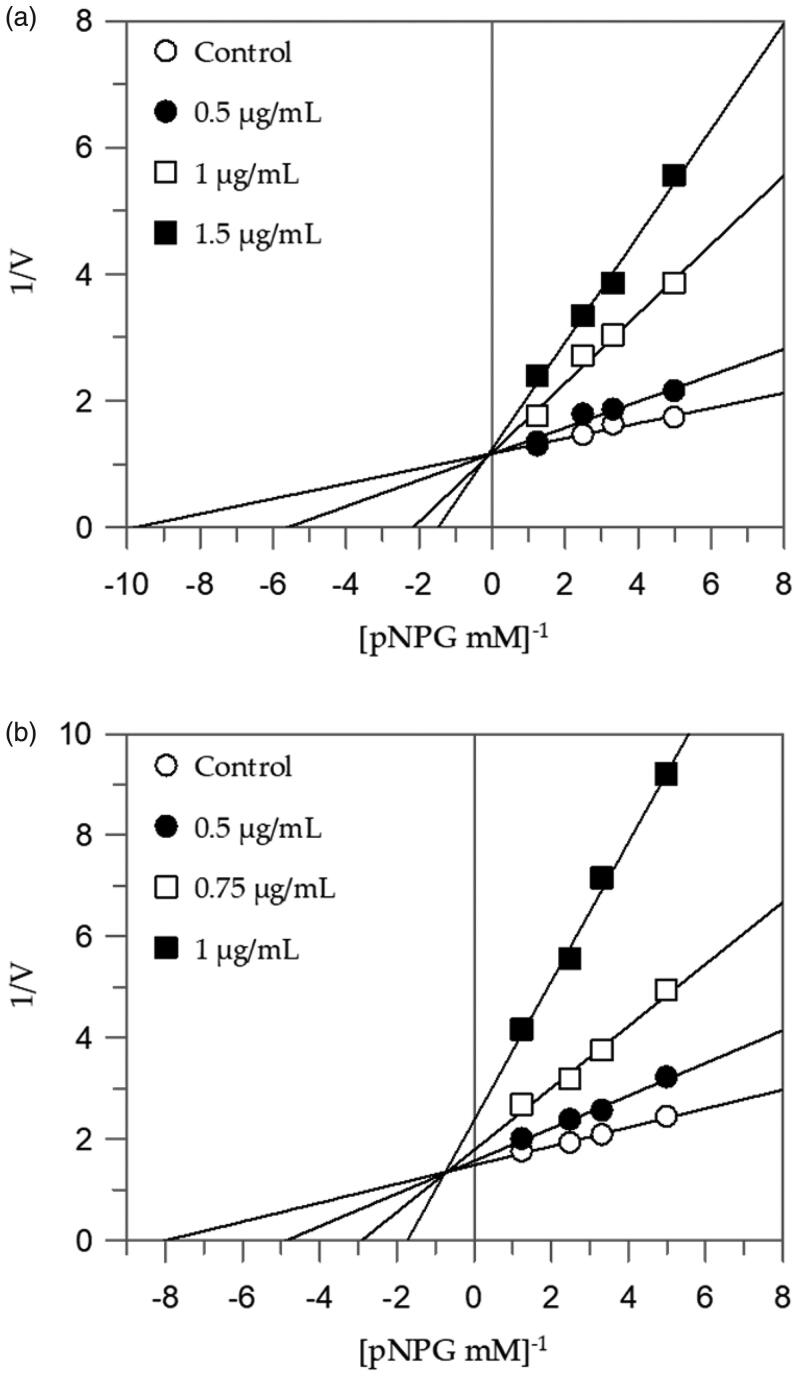
Inhibition of *α*-glucosidase enzyme. Lineweaver–Burk plots analysis of EEG (a) and MEG (b).

Table 2 shows that EEG acts as a competitive inhibitor against *α*-glucosidase. In fact, by increasing the concentration of extract, a family of straight lines with different slope, all intersecting on the *y*-axis, was found (Figure 1(a)). This kinetic analysis indicates that the extract binds with the free enzyme and the equilibrium constant, *K*_I_ = 0.08 µg/mL, was obtained from the slope (*K_m_*/*V*_max_) versus inhibitor concentration.

**Table 2.** Enzyme kinetics parameters following *α*-glucosidase and *α*-amylase with different *W. filifera* seed extracts.

	K_I_ µg/mL	K_IS_ µg/mL	Inhibition type
		*α-glucosidase*	
EEG	0.08	–	Competitive
EES	0.22	0.41	Mixed
MEG	0.08	1.8	Mixed
MES	0.03	0.48	Mixed
AEG	0.07	0.41	Mixed
AES	0.31	1.74	Mixed
		*α-amylase*	
EES	–	2.47	Uncompetitive
MES	–	3.66	Uncompetitive

The EES showed a different mode of inhibition if compared to the other alcoholic extract, although the composition of alcoholic extracts of the seeds is the same. The relative quantity of the compounds found in the extract could explain the consequent different type of inhibition.

The Lineweaver-Burk plots of the other extracts showed a mixed type of inhibition characteristics, since increasing the concentration of extracts resulted in a family of straight lines with different slope and y-intercepts, which intersected in the second quadrant, example in Figure 1(b). This kinetic analysis indicates that these extracts can bind not only with the free enzyme but also with the enzyme-substrate complex. The equilibrium constants for binding with the free enzyme (K_I_) and with the enzyme-substrate complex (*K_IS_*) were obtained from the slope (*K_m_*/*V*_max_) or the 1/*V*_max_ values (*y*-intercepts) versus inhibitor concentration, respectively (Table 2).

**Figure 2.**
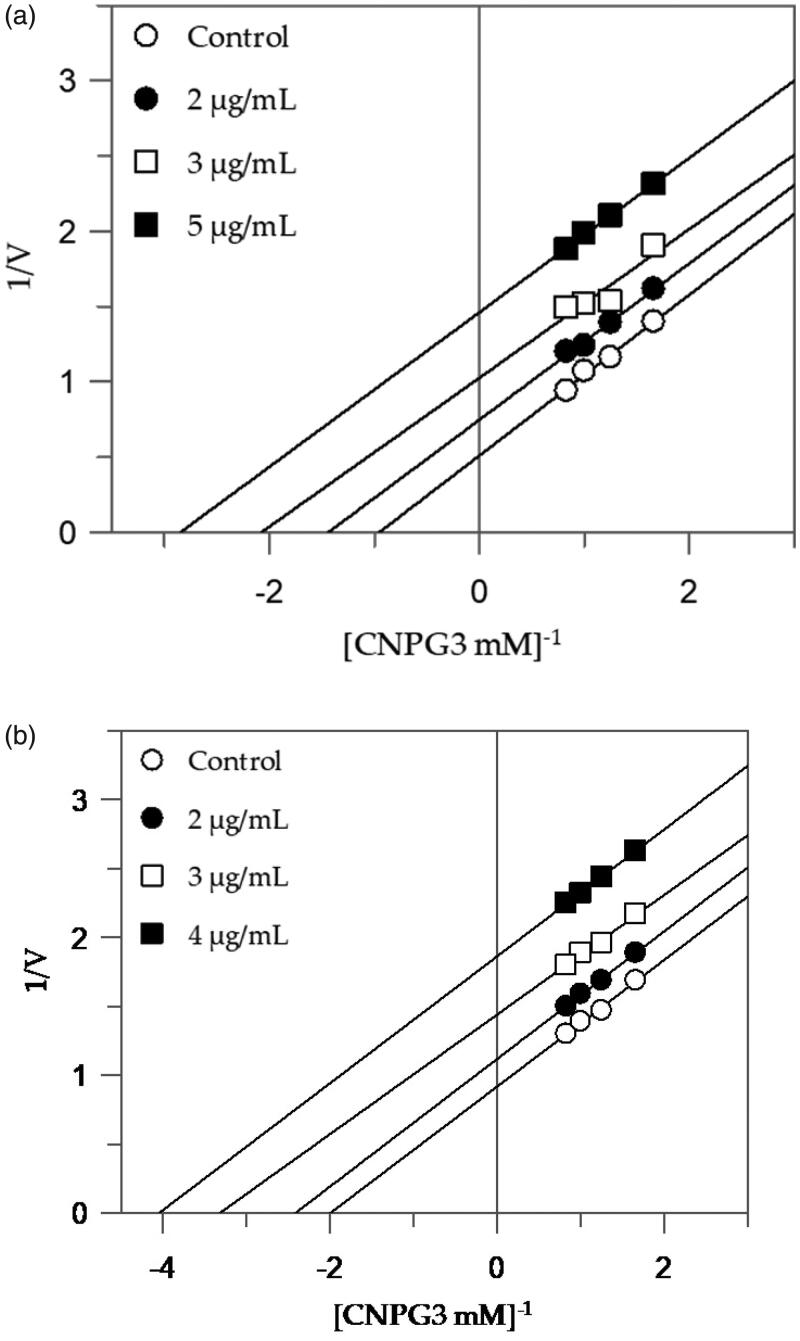
Inhibition of *α*-amylase enzyme. Lineweaver–Burk plots analysis of EES (a) and MES (b).

The extracts with higher inhibitory activity against *α*-amylase, EES, and MES, behave with an uncompetitive inhibition, since the kinetic analysis of these extracts, produces a family of parallel lines for increasing extracts concentration (Figures 2(a,b)). The equilibrium constant for binding with the enzyme-substrate complex (*K*_IS_) was calculated from the replotting of the intercepts (1/*V*_max_) versus the inhibitor concentration, resulting in a value of 2.47 and 3.66 µg/mL for EES and MES, respectively (Table 2).

*W. filifera* methanolic seeds extract from Sousse (MES) showed to be as the overall best extract, having the highest inhibitory activity against *α*-amylase and among the lowest IC_50_ values for *α*-glucosidase (100-fold lower than acarbose).

Moreover, acarbose is one of the therapeutic drugs used for the treatment of hyperglycaemia in T2D patients but several side effects generally occur, probably due to a more significant inhibition of *α*-amylase if compared to *α*-glucosidase inhibition^42^. MES instead showed a better inhibition against *α*-glucosidase showing also a lower ratio between *α*-glucosidase and *α*-amylase inhibition, making this extract as a good candidate for further deeper study. Thus, MES was tested for the ThT assay.

### Inhibition of IAPP aggregate formation

For the identification and quantification of amyloid fibrils *in vitro, e*xtrinsic fluorescence of the ThT was used to monitor fibrillation kinetics in real-time. When ThT binds to β-sheet-rich structures such as amyloid fibrils, ThT displays enhanced fluorescence and a characteristic blue shift in the emission spectrum when bound to amyloid fibrils. The corresponding spectra are measured, and the fluorescence intensities of the dye are plotted as a function of time. Any deviation from the control sample along the time scale, that is, peptide aggregation in the absence of any additive, could be indicative of inhibition or acceleration of the aggregation processes.

**Figure 3.**
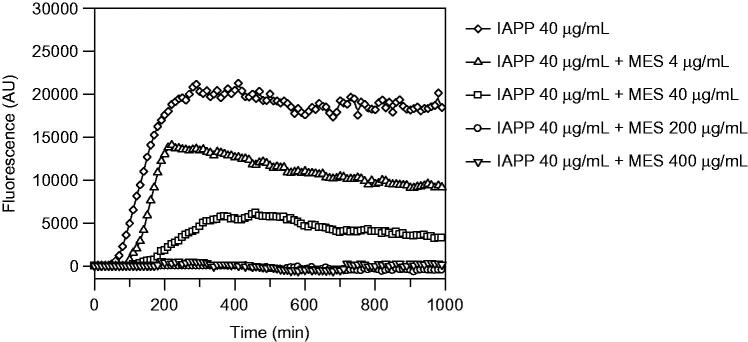
Thioflavin T fluorescence emission plot corresponding to β-sheet formation of IAPP in the presence of *W. filifera* methanolic seeds extract from Sousse.

The methanolic extract showed complete inhibition of fibrils formation at 1:5 (IAPP 40 µg/mL:MES 200 µg/mL) and 1:10 (IAPP 40 µg/mL:MES 400 µg/mL) ratio (Figure 3), in fact, the corresponding curves appear flat and superimposed, meaning that there is no formation of fibrils. There is also a delay at 1:1 (IAPP 40 µg/mL:MES 40 µg/mL) ratio on fibril formation, made evident by the shifting of the curve that is smaller by decreasing the concentration of the extract, highlighting that the inhibition is dose-dependent. The ThT assay was repeated three times and extended up to 150 h.

Congo red staining was performed to visualise the presence of amyloid fibrils. The cotton dye Congo red binds to amyloid fibrils, that appear red-orange. When observed with crossed polarisers in the polarisation microscopy, stained amyloid exhibits bright green birefringence, often referred to as “apple green birefringence”. Congo red staining of solution 1:5 (IAPP 40 µg/mL:MES 200 µg/mL) was negative and confirmed that inhibition of IAPP fibrillation was occurred.

**Figure 4.**
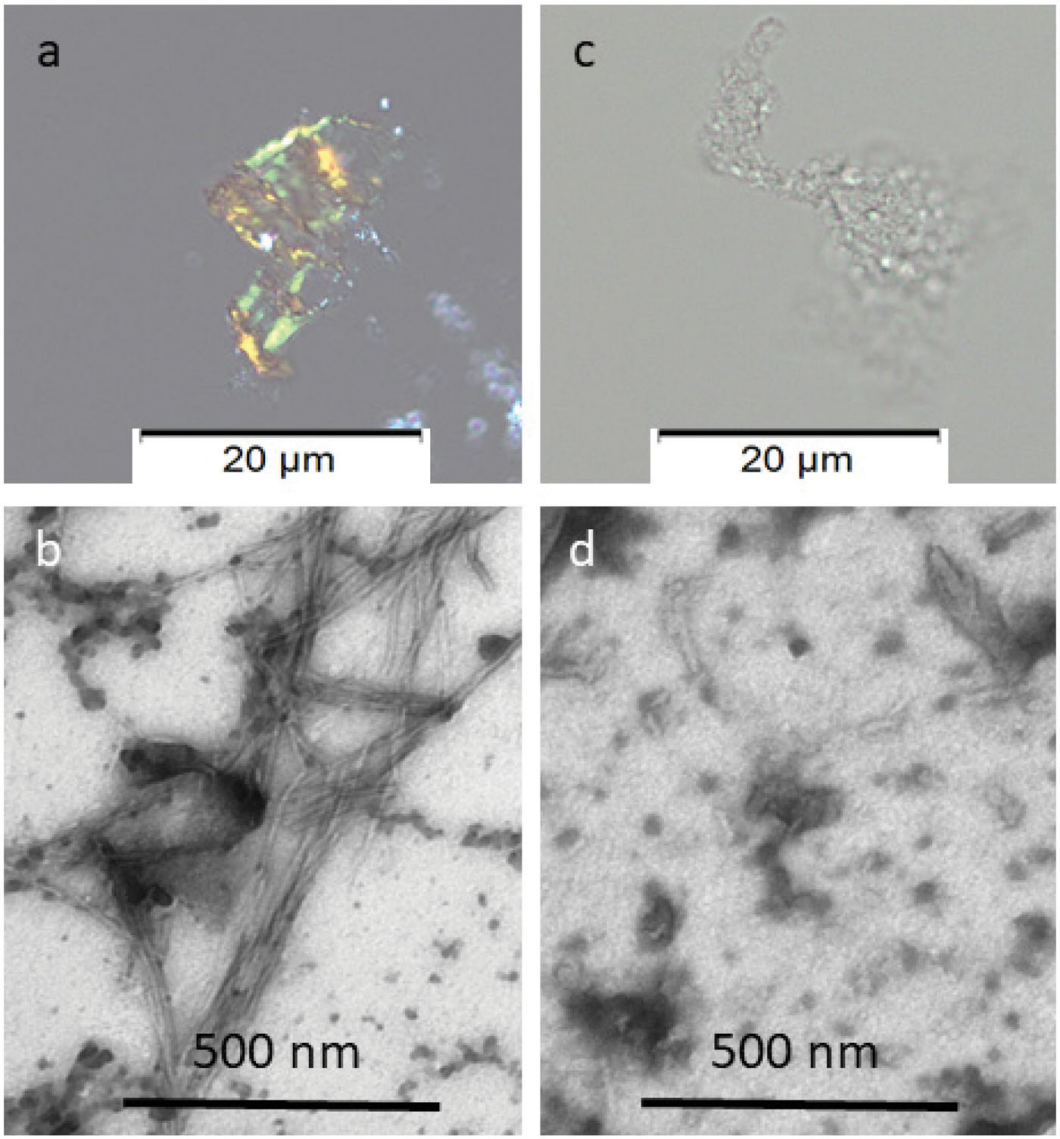
Congo red and Electron microscopy analyses of the material recovered after ThT analysis. In (a), amyloid exhibiting green birefringence after Congo red staining and (b) long unbranched amyloid fibrils are present in solution of IAPP 40 µg/mL. In (c), no Congophilc material can be detected and in (d) an amorphous material is present in solution containing IAPP 40 µg/mL with MES 200 µg/mL. Samples in a and c are stained with Congo red and samples in b and d are negatively contrasted with 2% Uranyl acetate in 50% ethanol.

In addition, negative staining was made for analysis by Transmission Electron Microscopy (TEM), a useful technique for assessing the morphology of *in vitro* formed amyloid fibrils from proteins or peptides that allows researchers to see structural features at the nanometre scale that cannot be visualised by light microscopy. Negative staining typically generates the sample with good contrast and well-preserved morphology. The stain forms a coating over the sample that appears light and the surrounding stain appears dark. Figure 4 showed that at 1:5 IAPP to extract ratio (MES 200 µg/mL), the formation of IAPP fibrils is inhibited.

### Molecular docking

In the present study, as a first step, molecular docking of the main compounds identified in methanolic seed extract of *W. filifera* was performed against α-glucosidase, α-amylase and IAPP to determine their binding affinities. The binding affinity data of the compounds is presented in Table 3.

**Table 3.** Binding energies of against IAPP, α-amylase and α-glucosidase.

	IAPP	α-amylase	α-glucosidase
Catechin	−6.9 kcal/mol	−7.1 kcal/mol	−8.1 kcal/mol
Protocatechuic acid	−4.9 kcal/mol	−5.5 kcal/mol	−5.3 kcal/mol
*p*-Hydroxybenzoic acid	−4.5 kcal/mol	−5.2 kcal/mol	−5.2 kcal/mol
B type Procyanidin dimer	−7.8 kcal/mol	−8.5 kcal/mol	−4.9 kcal/mol

The binding affinity of the compounds ranges from −4.5 to −7.8 kcal/mol towards IAPP, from −5.2 kcal/mol to −8.5 kcal/mol for the α-amylase and ranging from −4.9 kcal/mol to −8.1 kcal/mol regarding α-glucosidase. Among the four compounds, catechin displayed better binding energy values for all the three protein targets.

An investigation of the physicochemical properties of the main compounds identified in this extract is reported in Table 4.

**Table 4.** Physicochemical properties of bioactive compounds in the investigated extracts.

	Molecular formula	Molecular Weight (g/mol)	Rotatable bonds	H-bond acceptor atoms	H-bond donor atoms	Polar surface area (Å^2^)	Log*P*_o/w_	Water solubility
Catechin	C15H14O6	290.27	1	6	5	110.38	0.85	Soluble
Epicatechin	C15H14O6	290.27	1	6	5	110.38	0.85	Soluble
Protocatechuic acid	C7H6O4	154.12	1	4	3	77.76	0.65	Soluble
*p*-Hydroxybenzoic acid	C7H6O3	138.12	1	3	2	57.53	1.05	Soluble
Procyanidin B-type	C30H26O12	578.52	3	12	10	220.76	1.37	Soluble
Procyanidin A-type	C30H24O12	576.50	2	12	9	209.76	1.61	Moderately
Quercetin-3-O-glucoside	C21H20O12	464.38	4	12	8	210.51	−0.27	Soluble
Dihydrokaempferol 7-glucoside	C21H22O11	450.39	4	11	7	186.37	−0.48	Soluble

## Conclusions

Controlling the response of carbohydrate hydrolysing enzymes (*α*-amylase and *α*-glycosidase) is an effective strategy in facing and/or managing postprandial hyperglycaemia. *α*-Amylase is endoglucanase, which hydrolyses the internal α-1,4 glycosidic linkage in starch and *α*-glucosidase is one of the glucosidases located in the brush border surface membrane of intestinal cells, key enzyme for carbohydrate digestion.

The results of this study showed that *W. filifera* extracts had significant inhibitory activities against *α*-amylase, *α*-glucosidase. In addition to these properties, the methanolic *W. filifera* seed extract has shown a complete inhibition of the formation of the toxic IAPP aggregates. Since such aggregates are implicate in pancreatic β-islet cell death, inhibition of IAPP aggregation is important in prevention of diabetes or of its progression.

To better understand the potential of the methanolic extract, further studies are needed to know the role of the compounds and their possible synergic action in the inhibition of α-amylase, α-glucosidase and the IAPP fibril formation, key targets of diabetes.

Considering our previous data obtained on the effect of *W. filifera* seed extracts on target enzymes for AD and the correlation between AD and T2D, *W. filifera* seeds can emerge as a promising natural source of bioactive compounds for these diseases.

Moreover, our results are of interest considering that seeds are an inedible part of the fruit that is discarded.

## References

[1] Tan Y, Chang SKC, Zhang Y. Comparison of α-amylase, α-glucosidase and lipase inhibitory activity of the phenolic substances in two black legumes of different genera. Food Chem 2017;214:259–68. 27507474 10.1016/j.foodchem.2016.06.100

[2] Kaku K. Efficacy of voglibose in type 2 diabetes. Expert Opin Pharmacother 2014;15:1181–90. 24798092 10.1517/14656566.2014.918956

[3] Mata R, Cristians S, Escandon-Rivera S, et al. Mexican antidiabetic herbs: valuable sources of inhibitors of α-glucosidases. J Nat Prod 2013;76:468–83. 23398496 10.1021/np300869g

[4] Kajaria D, Ranjana Tripathi J, Tripathi YB, et al. In-vitro alpha amylase and glycosidase inhibitory effect of ethanolic extract of antiasthmatic drug – shirishadi. J Adv Pharm Technol Res 2013;4:206–9. 24350051 10.4103/2231-4040.121415 PMC3853697

[5] Loizzo MR, Marrelli M, Pugliese A, et al. Crocus cancellatus subsp. Damascenus stigmas: chemical profile, and inhibition of α-amylase, α-glucosidase and lipase, key enzymes related to type 2 diabetes and obesity. J Enzyme Inhib Med Chem 2016;31:212–8. 25792502 10.3109/14756366.2015.1016510

[6] Majeed M, Majeed S, Mundkur L, et al. Standardized emblica officinalis fruit extract inhibited the activities of α-amylase, α-glucosidase, and dipeptidyl peptidase-4 and displayed antioxidant potential. J Sci Food Agric 2020;100:509–16. 31487036 10.1002/jsfa.10020 PMC6973029

[7] Pan CY. Reducing the risk of type 2 diabetes: early identification of high-risk individuals and treatment with acarbose. Curr Diabetes Rev 2007;3:141–8. 18220665 10.2174/157339907780598243

[8] Yin Z, Zhang W, Feng F, et al. Α-glucosidase inhibitors isolated from medicinal plants. Food Sci Hum Wellness 2014;3:136–74.

[9] Gad MZ, El-Sawalhi MM, Ismail MF, El-Tanbouly ND. Biochemical study of the anti-diabetic action of the egyptian plants fenugreek and balanites. Mol Cell Biochem 2006;281:173–83. 16328970 10.1007/s11010-006-0996-4

[10] Ashrafizadeh M, Najafi M, Orouei S, et al. Resveratrol modulates transforming growth factor-beta (tgf-β) signaling pathway for disease therapy: a new insight into its pharmacological activities. Biomedicines 2020;8:261. 10.3390/biomedicines8080261 PMC7460084 32752069

[11] Etxeberria U, de la Garza AL, Campión J, et al. Antidiabetic effects of natural plant extracts via inhibition of carbohydrate hydrolysis enzymes with emphasis on pancreatic alpha amylase. Exp Opin Ther Targets 2012;16:269–97. 10.1517/14728222.2012.664134 22360606

[12] Sajid M, Khan MR, Ismail H, et al. Antidiabetic and antioxidant potential of *Alnus nitida* leaves in alloxan induced diabetic rats. J Ethnopharmacol 2020;251:(112544. 31904496 10.1016/j.jep.2020.112544

[13] Bindu J, Narendhirakannan RT. Role of medicinal plants in the management of diabetes mellitus: a review. 3 Biotech 2019;9:4. 10.1007/s13205-018-1528-0 PMC6291410 30555770

[14] Lee BW, Ha TKQ, Pham HTT, et al. Hydroxyoleoside-type seco-iridoids from symplocos cochinchinensis and their insulin mimetic activity. Sci Rep 2019;9:2270. 30783120 10.1038/s41598-018-38013-4 PMC6381099

[15] Chuengsamarn S, Rattanamongkolgul S, Luechapudiporn R, et al. Curcumin extract for prevention of type 2 diabetes. Diabetes Care 2012;35:2121–7. 22773702 10.2337/dc12-0116 PMC3476912

[16] Mahmoud AM, Ashour MB, Abdel-Moneim A, Ahmed OM. Hesperidin and naringin attenuate hyperglycemia-mediated oxidative stress and proinflammatory cytokine production in high fat fed/streptozotocin-induced type 2 diabetic rats. J Diabetes Complications 2012;26:483–90. 22809898 10.1016/j.jdiacomp.2012.06.001

[17] Atawodi SE. Antioxidant potential of african medicinal plants. Afr J Biotechnol 2005;4:128–33.

[18] Westermark P, Andersson A, Westermark GT. Islet amyloid polypeptide, islet amyloid, and diabetes mellitus. Physiol Rev 2011;91:795–826. 21742788 10.1152/physrev.00042.2009

[19] Westermark P. Quantitative studies on amyloid in the islets of Langerhans. Upsala J Med Sci 1972;77:91–4. 4116019 10.1517/03009734000000014

[20] Westermark GT, Krogvold L, Dahl-Jørgensen K, Ludvigsson J. Islet amyloid in recent-onset type 1 diabetes-the DiViD study. Upsala J Med Sci 2017;122:201–3. 28814132 10.1080/03009734.2017.1359219 PMC5649327

[21] Butler AE, Janson J, Soeller WC, Butler PC. Increased beta-cell apoptosis prevents adaptive increase in beta-cell mass in mouse model of type 2 diabetes: evidence for role of islet amyloid formation rather than direct action of amyloid. Diabetes 2003;52:2304–14. 12941770 10.2337/diabetes.52.9.2304

[22] Lorenzo A, Razzaboni B, Weir GC, Yankner BA. Pancreatic islet cell toxicity of amylin associated with type-2 diabetes mellitus. Nature 1994;368:756–60. 8152488 10.1038/368756a0

[23] Madhusudhanan J, Suresh G, Devanathan V. Neurodegeneration in type 2 diabetes: Alzheimer’s as a case study. Brain Behav 2020;10:e01577. 32170854 10.1002/brb3.1577 PMC7218246

[24] Geavlete P, Multescu R, Geavlete B. *Serenoa repens* extract in the treatment of benign prostatic hyperplasia. Ther Adv Urol 2011;3:193–8. 21969849 10.1177/1756287211418725 PMC3175703

[25] Nehdi IA. Characteristics and composition of *Washingtonia filifera* (linden ex andré) h. Wendl. Seed and seed oil. Food Chem 2011;126:197–202.

[26] Cornett JW. Nutritional value of desert fan palm fruits. Principes 1987;31:159–61.

[27] Floris S, Fais A, Rosa A, et al. Phytochemical composition and the cholinesterase and xanthine oxidase inhibitory properties of seed extracts from the *Washingtonia filifera* palm fruit. RSC Adv 2019;9:21278–87. 10.1039/c9ra02928a PMC9066185 35521327

[28] Fais A, Era B, Di Petrillo A, et al. Selected enzyme inhibitory effects of euphorbia characias extracts. BioMed Res Int 2018;2018:1219367–9. 30003087 10.1155/2018/1219367 PMC5996446

[29] Nanga RPR, Brender JR, Vivekanandan S, Ramamoorthy A. Structure and membrane orientation of iapp in its natively amidated form at physiological ph in a membrane environment. Biochim Biophys Acta (BBA) – Biomembr 2011;1808:2337–42. 10.1016/j.bbamem.2011.06.012 PMC3156962 21723249

[30] Bompard-Gilles C, Rousseau P, Rougé P, Payan F. Substrate mimicry in the active center of a mammalian alpha-amylase: structural analysis of an enzyme-inhibitor complex. Structure 1996;4:1441–52. 8994970 10.1016/s0969-2126(96)00151-7

[31] Waterhouse A, Bertoni M, Bienert S, et al. Swiss-model: homology modelling of protein structures and complexes. Nucl Acids Res 2018;46:W296–W303. 29788355 10.1093/nar/gky427 PMC6030848

[32] Yamamoto K, Miyake H, Kusunoki M, Osaki S. Crystal structures of isomaltase from saccharomyces cerevisiae and in complex with its competitive inhibitor maltose. FEBS J 2010;277:4205–14. 20812985 10.1111/j.1742-4658.2010.07810.x

[33] Luthra T, Banothu V, Adepally U, et al. Discovery of novel pyrido-pyrrolidine hybrid compounds as alpha-glucosidase inhibitors and alternative agent for control of type 1 diabetes. Eur J Med Chem 2020;188:112034. 31927314 10.1016/j.ejmech.2020.112034

[34] Benkert P, Biasini M, Schwede T. Toward the estimation of the absolute quality of individual protein structure models. Bioinformatics 2011;27:343–50. 21134891 10.1093/bioinformatics/btq662 PMC3031035

[35] O’Boyle NM, Banck M, James CA, et al. Open babel: an open chemical toolbox. J Cheminform 2011;3:33. 21982300 10.1186/1758-2946-3-33 PMC3198950

[36] Kumar A, Baccoli R, Fais A, et al. Substitution effects on the optoelectronic properties of coumarin derivatives. Appl Sci 2019;10:144.

[37] Fais A, Era B, Asthana S, et al. Coumarin derivatives as promising xanthine oxidase inhibitors. Int J Biol Macromol 2018;120:1286–93. 30189275 10.1016/j.ijbiomac.2018.09.001

[38] Wu Q, Peng Z, Zhang Y, Yang J. Coach-d: improved protein-ligand binding sites prediction with refined ligand-binding poses through molecular docking. Nucl Acids Res 2018;46:W438–42. 29846643 10.1093/nar/gky439 PMC6030866

[39] Dai T, Chen J, McClements DJ, et al. Investigation the interaction between procyanidin dimer and α-glucosidase: spectroscopic analyses and molecular docking simulation. Int J Biol Macromol 2019;130:315–22. 30794902 10.1016/j.ijbiomac.2019.02.105

[40] Zhang B, Deng Z, Ramdath DD, et al. Phenolic profiles of 20 Canadian lentil cultivars and their contribution to antioxidant activity and inhibitory effects on α-glucosidase and pancreatic lipase. Food Chem 2015;172:862–72. 25442631 10.1016/j.foodchem.2014.09.144

[41] Ho GTT, Kase ET, Wangensteen H, Barsett H. Phenolic elderberry extracts, anthocyanins, procyanidins, and metabolites influence glucose and fatty acid uptake in human skeletal muscle cells. J Agric Food Chem 2017;65:2677–85. 28303711 10.1021/acs.jafc.6b05582

[42] Oboh G, Ogunsuyi OB, Ogunbadejo MD, Adefegha SA. Influence of gallic acid on α-amylase and α-glucosidase inhibitory properties of acarbose. J Food Drug Anal 2016;24:627–34. 28911570 10.1016/j.jfda.2016.03.003 PMC9336674

